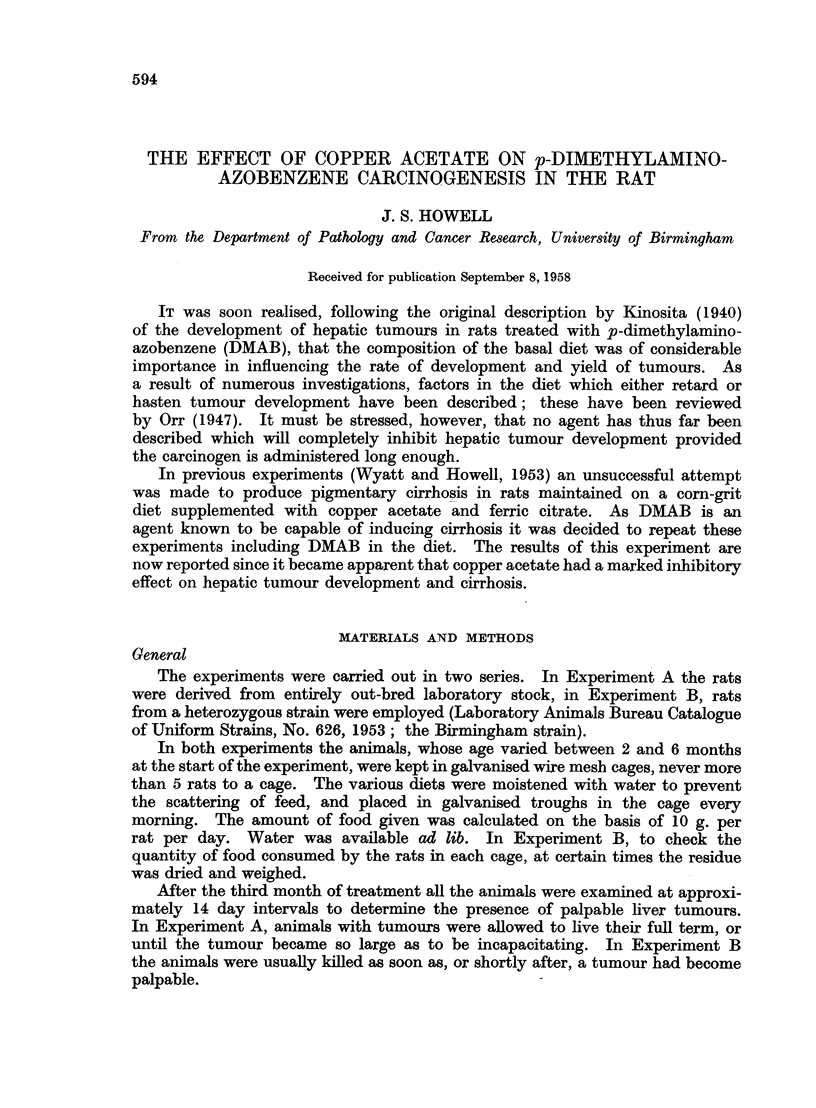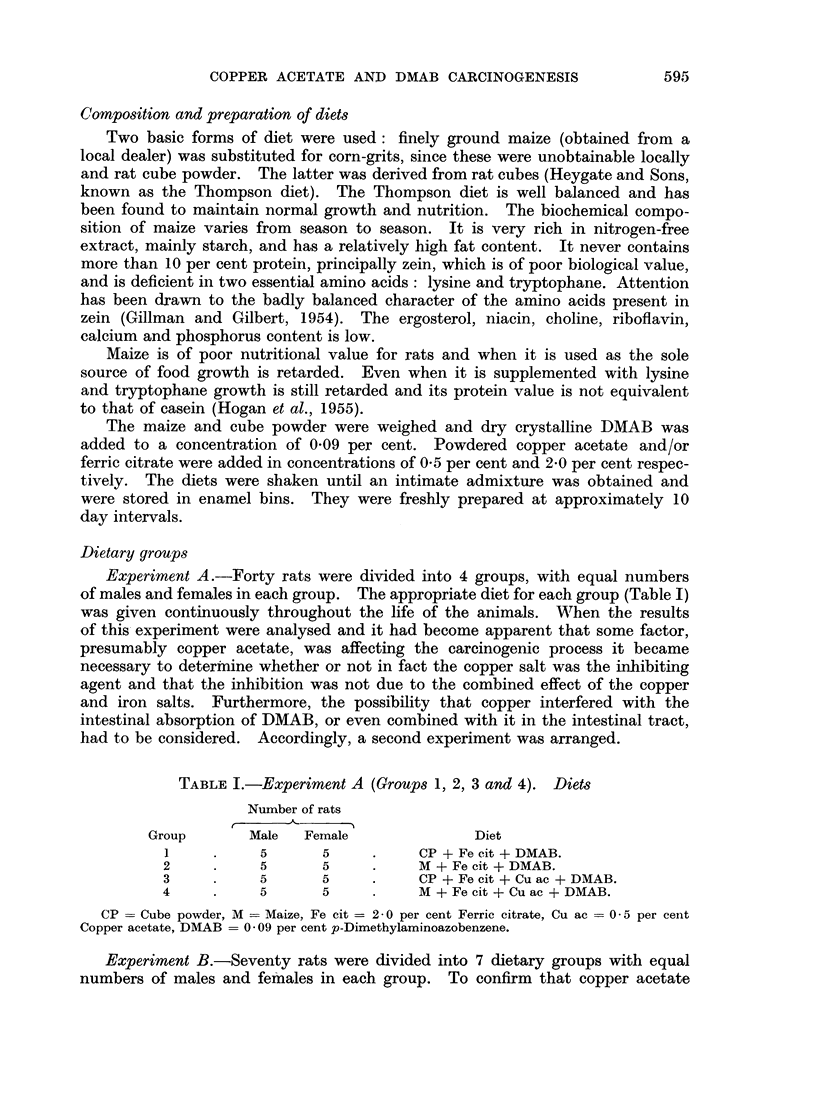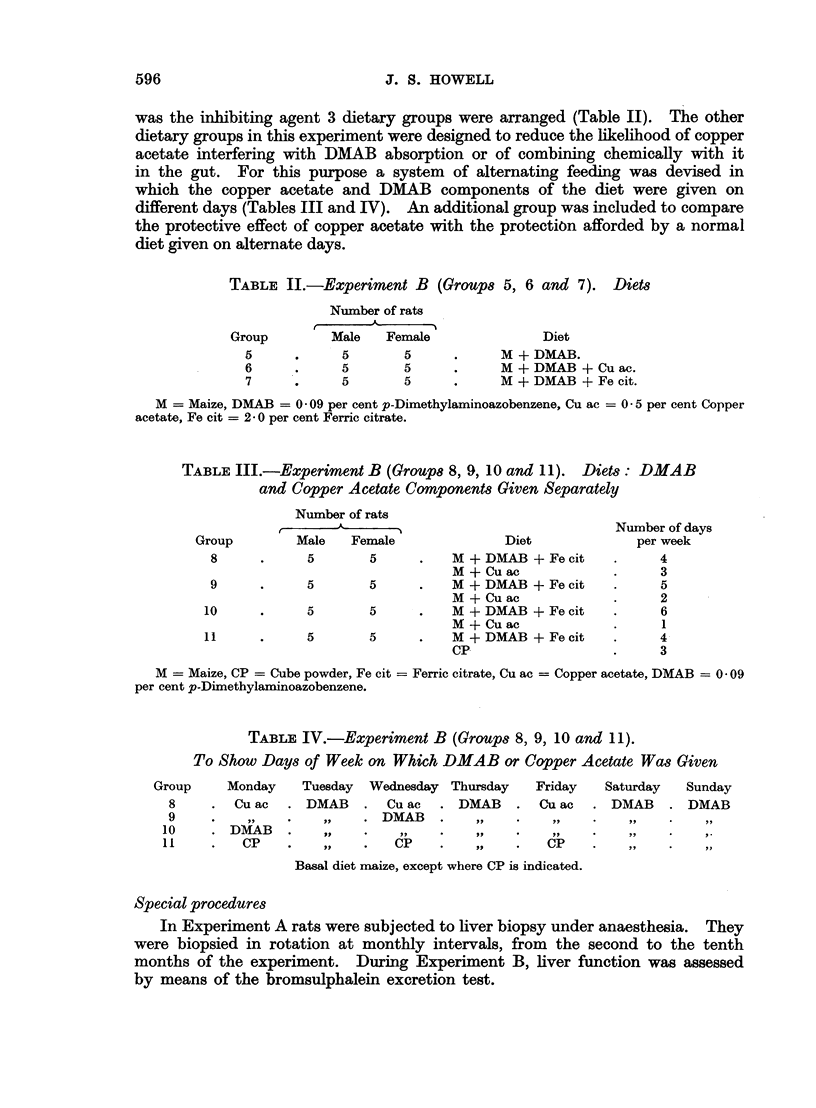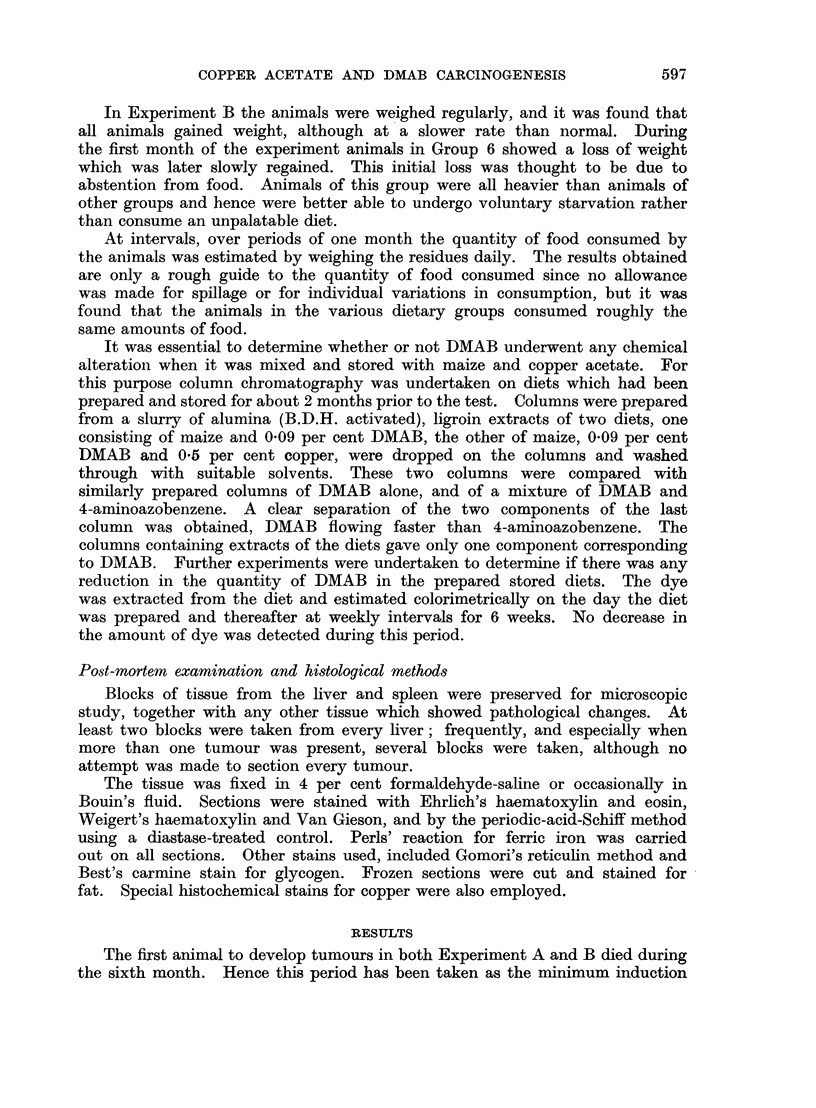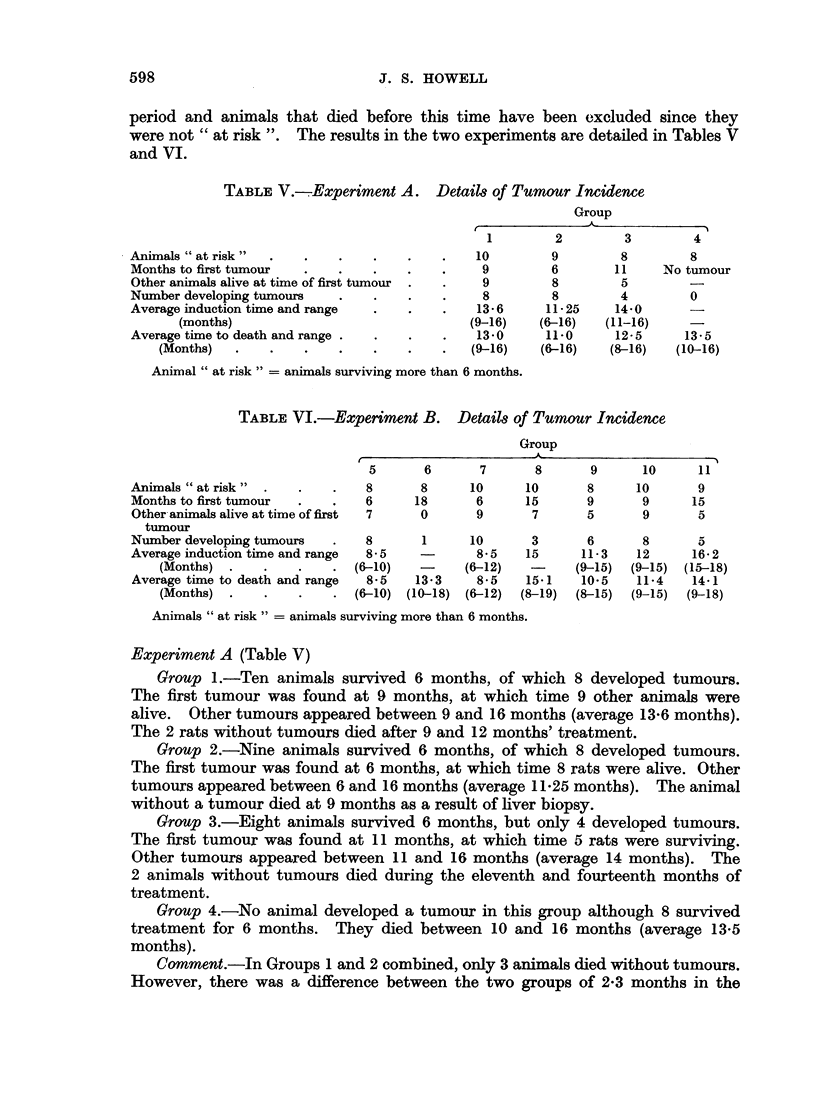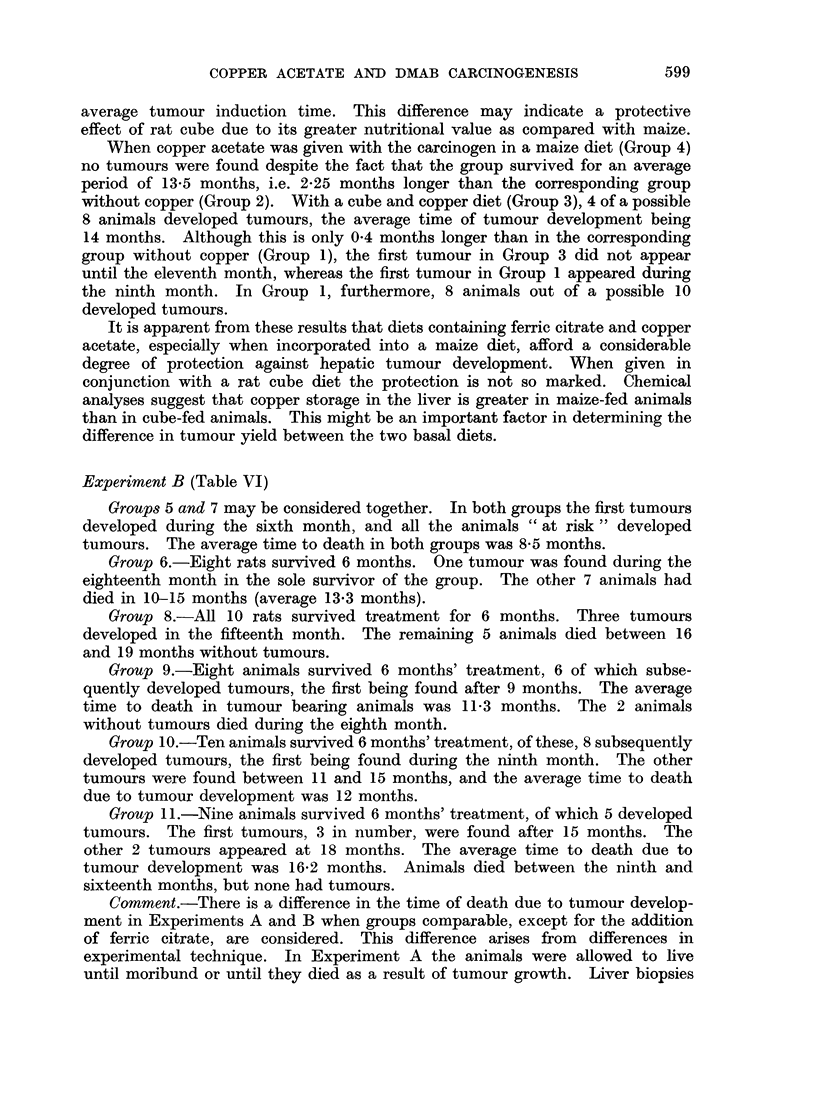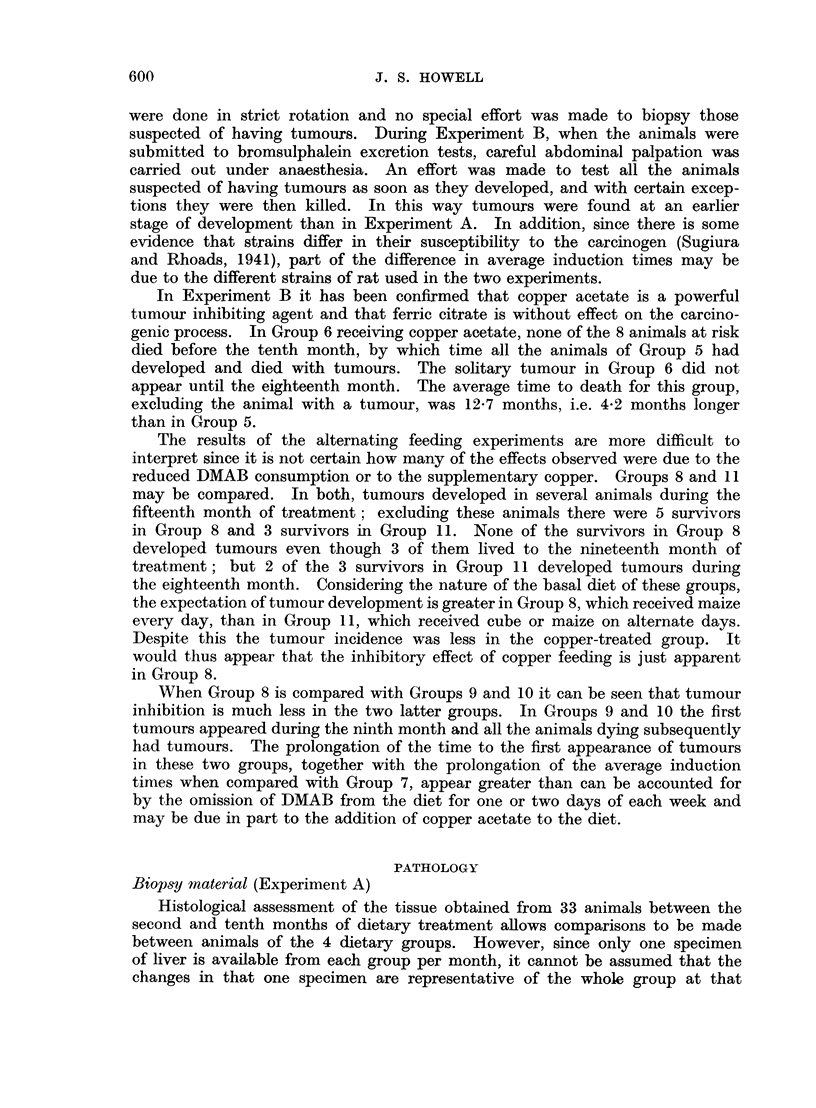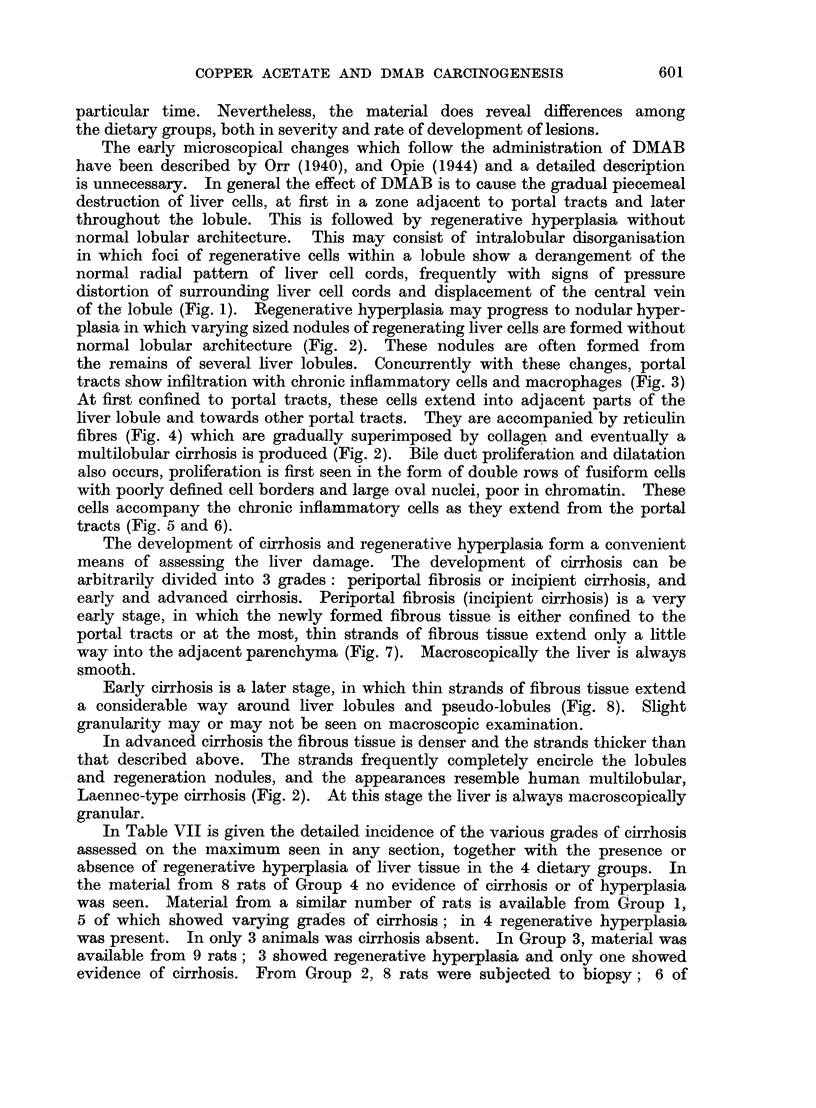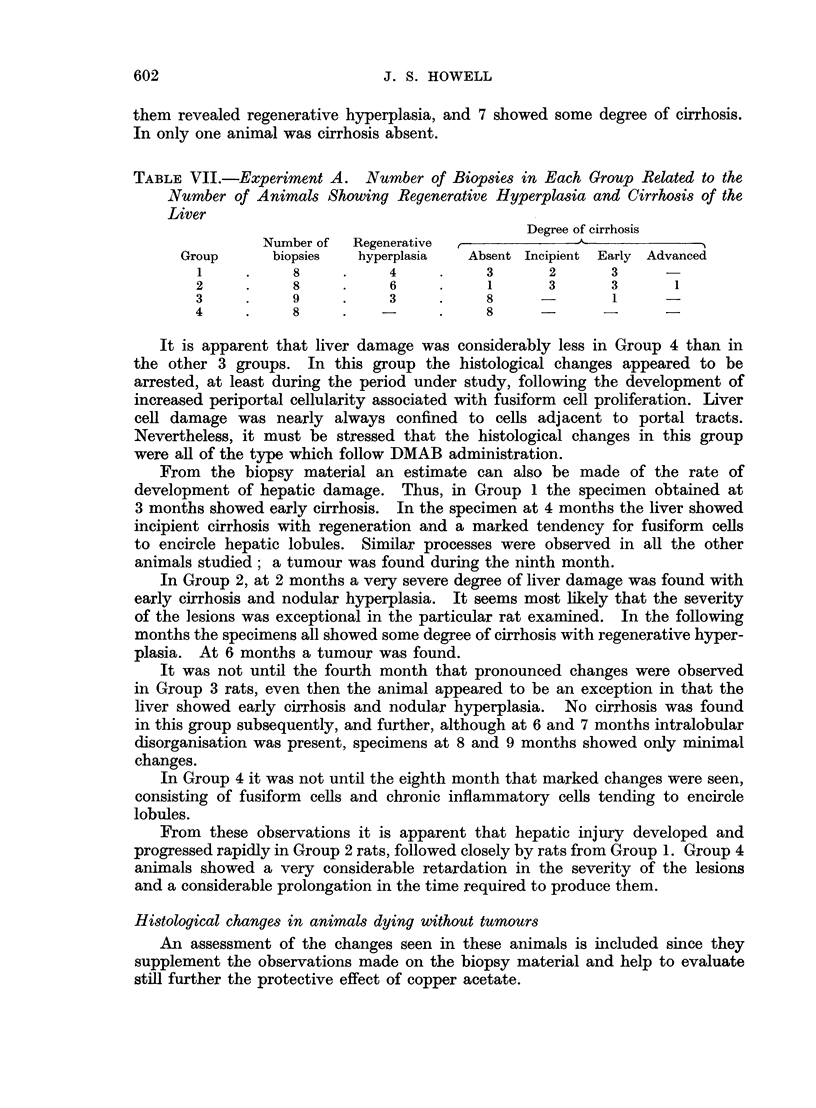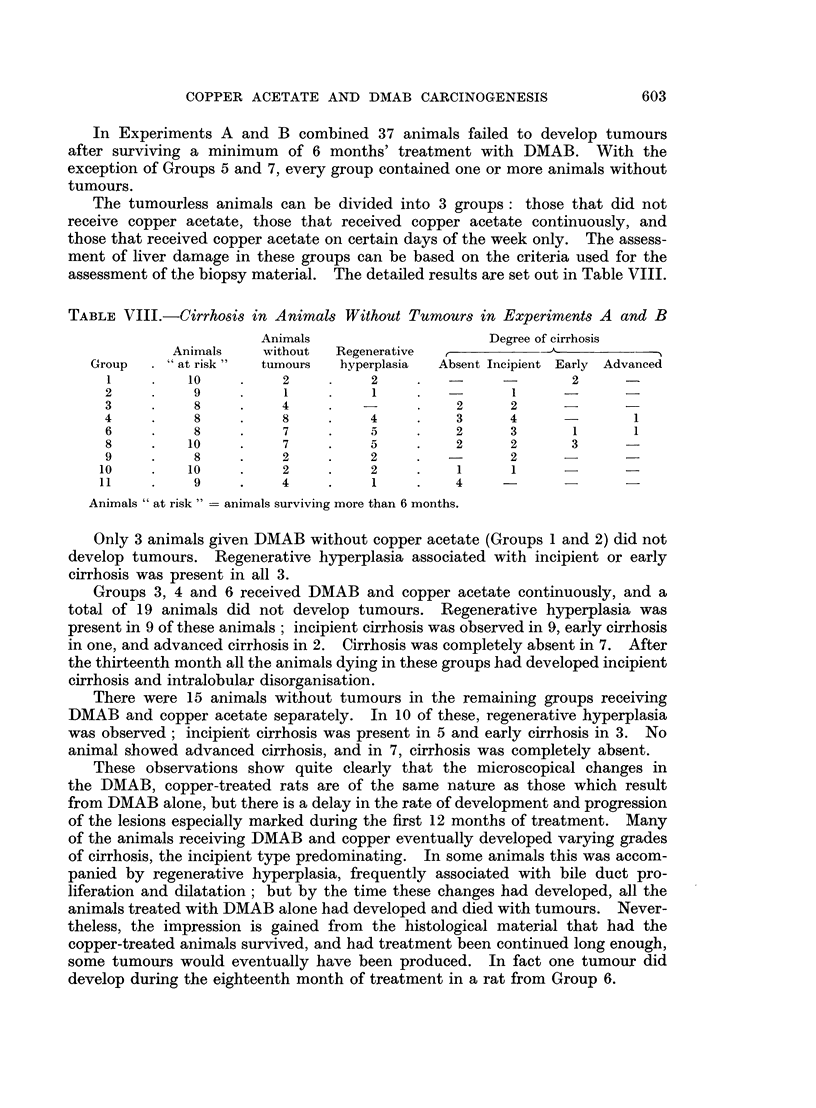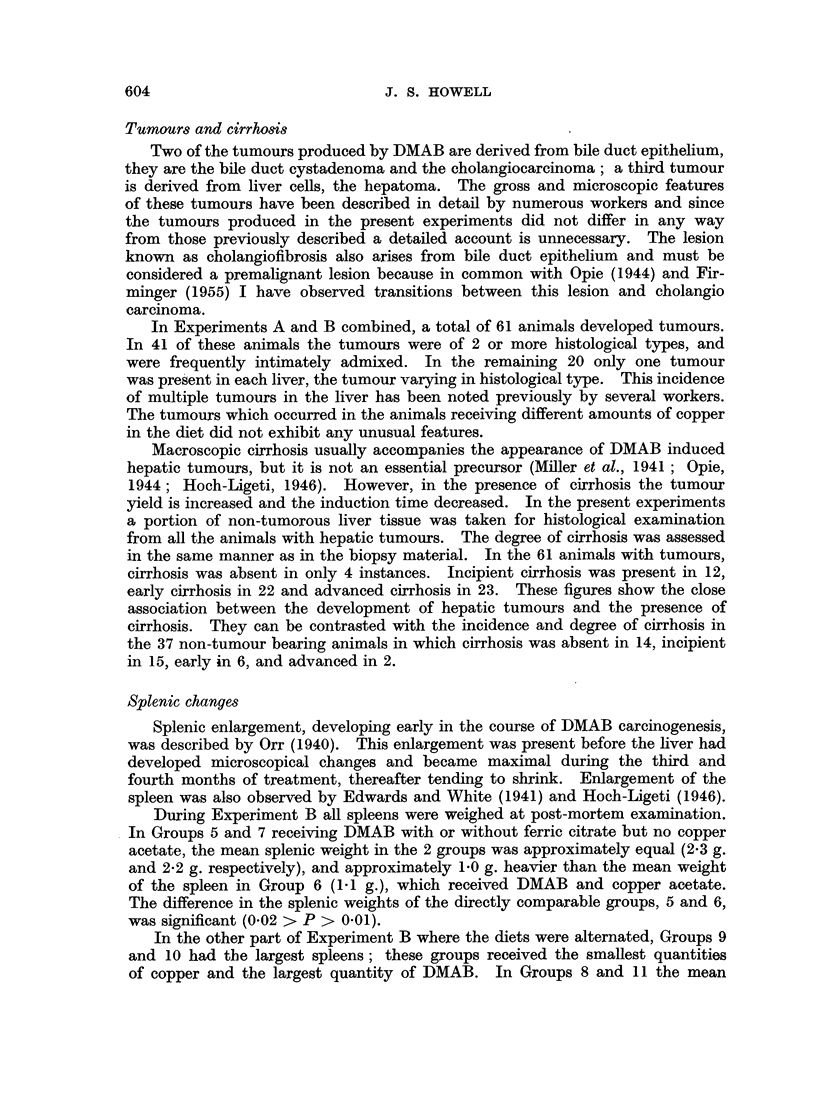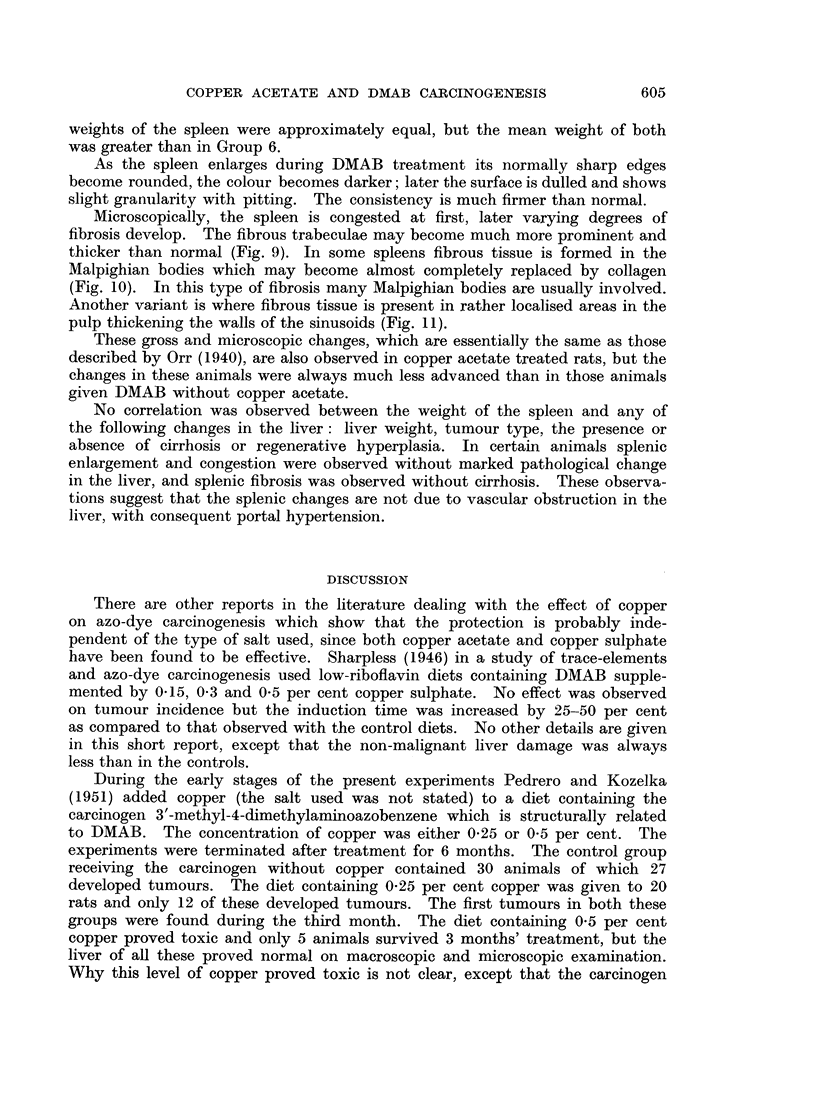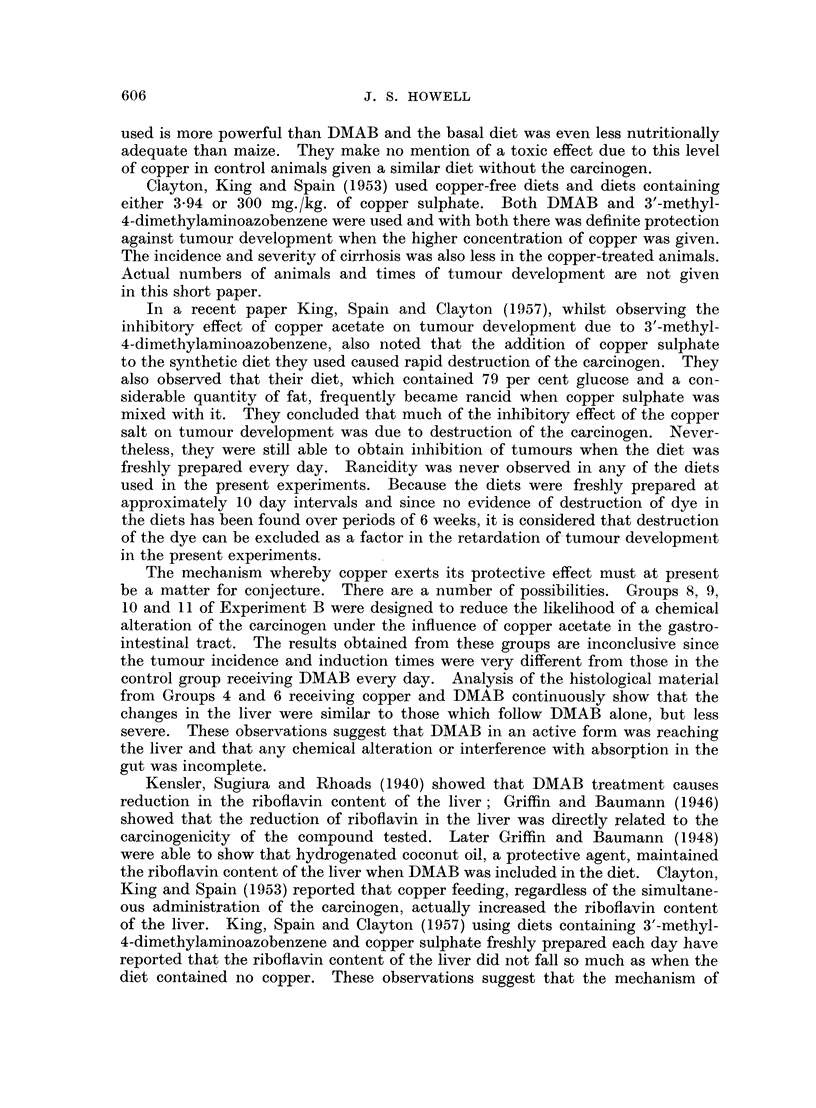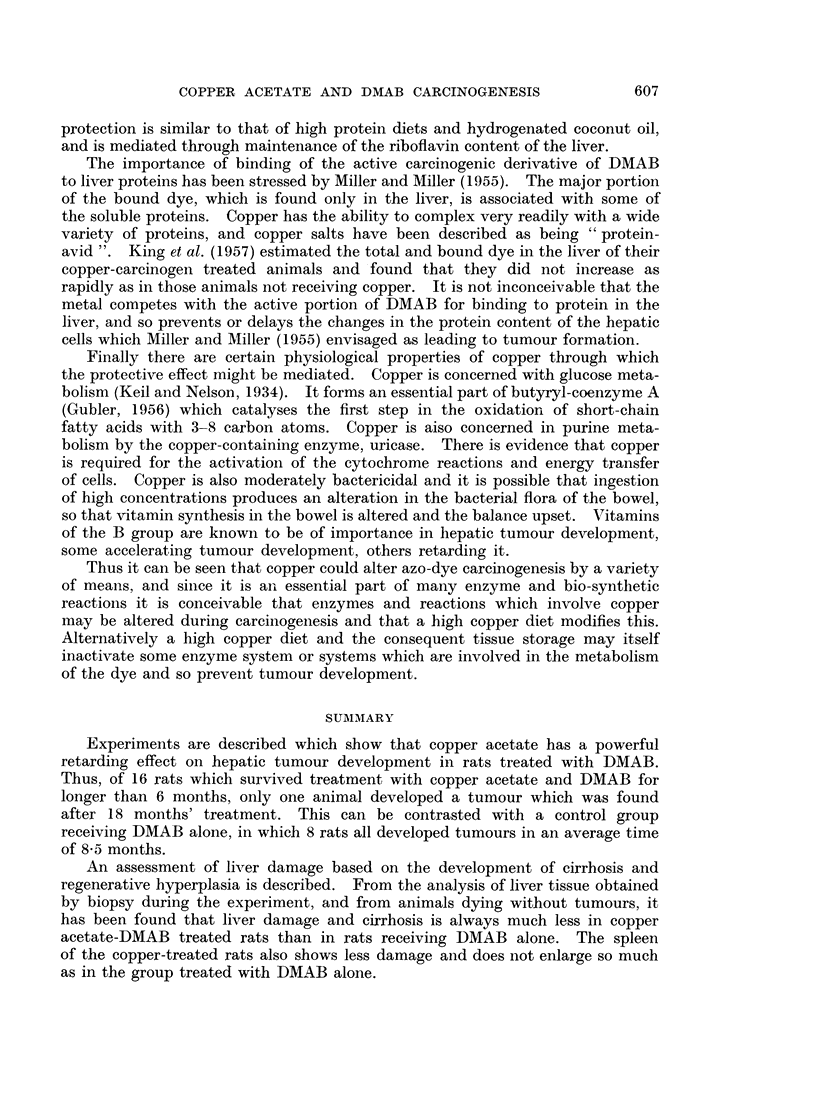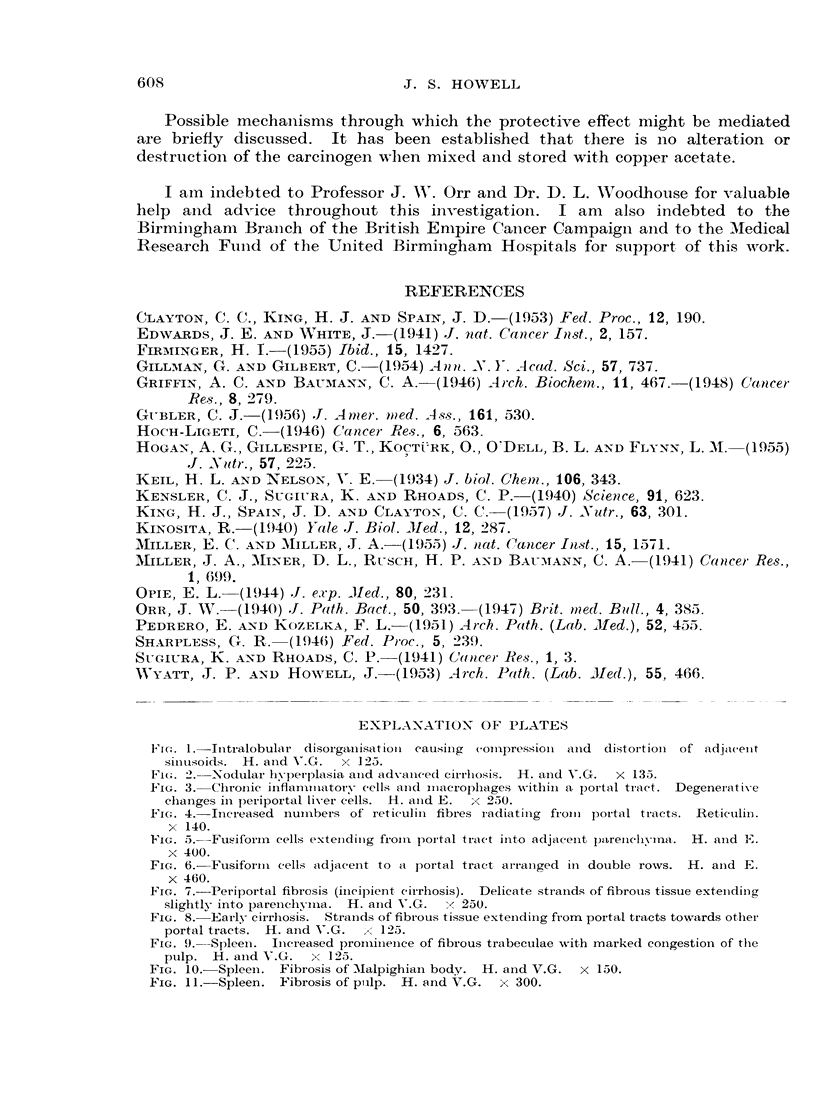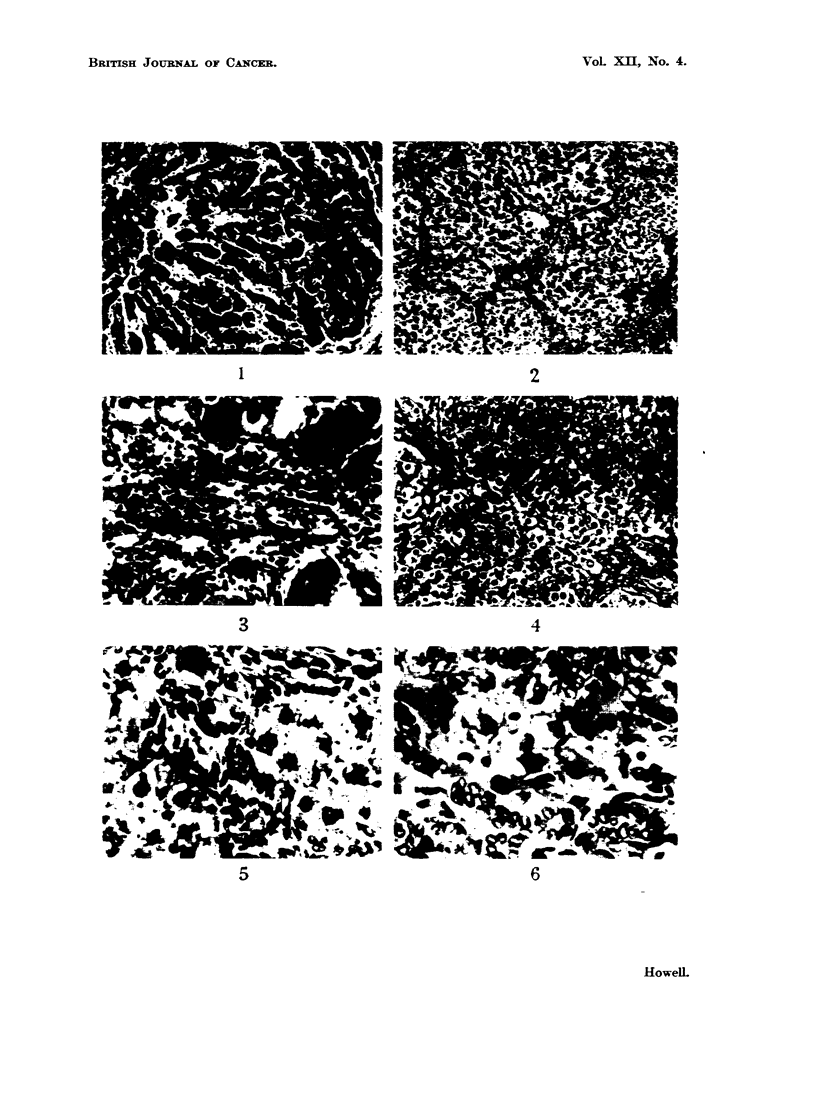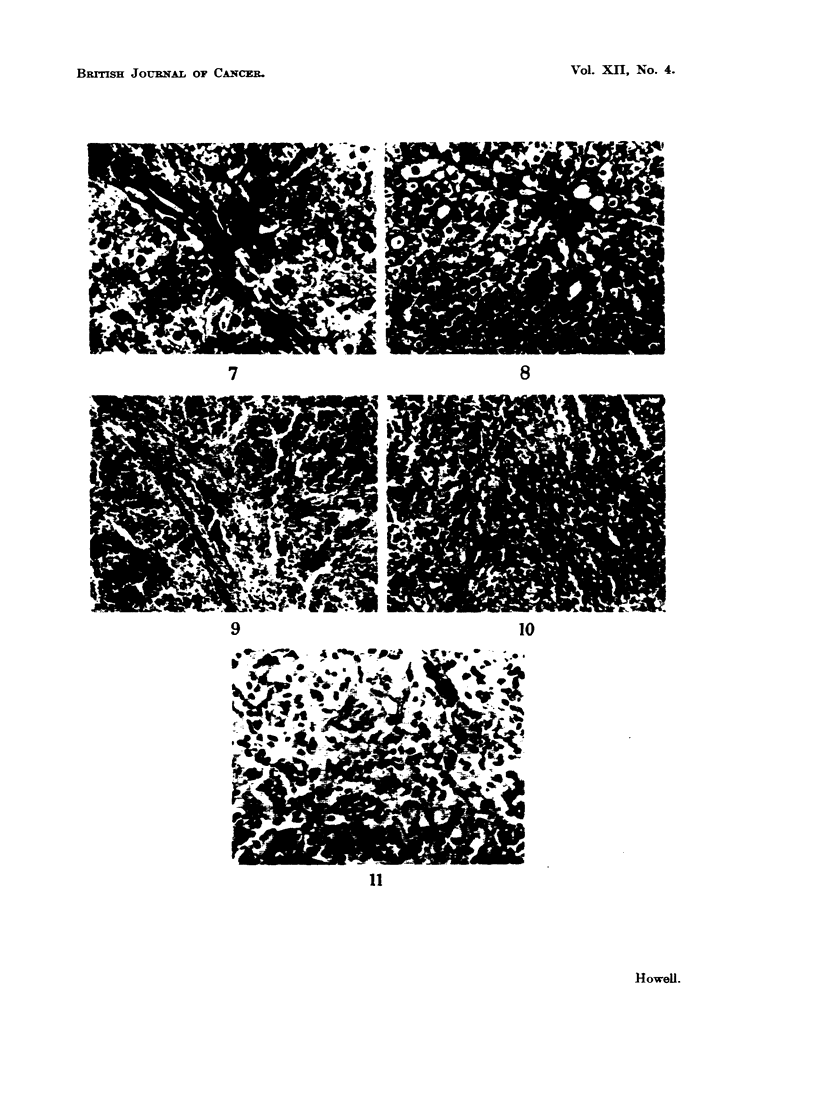# The Effect of Copper Acetate on p-Dimethylaminoazobenzene Carcinogenesis in the Rat

**DOI:** 10.1038/bjc.1958.67

**Published:** 1958-12

**Authors:** J. S. Howell

## Abstract

**Images:**


### THE EFFECT OF COPPER ACETATE ON *p*-DIMETHYLAMINO-AZOBENZENE CARCINOGENESIS IN THE RAT

J. S. HOWELL

*From the Department of Pathology and Cancer Research, University of Birmingham*

Received for publication September 8, 1958

IT was soon realised, following the original description by Kinosita (1940) of the development of hepatic tumours in rats treated with *p*-dimethylamino-azobenzene (DMAB), that the composition of the basal diet was of considerable importance in influencing the rate of development and yield of tumours. As a result of numerous investigations, factors in the diet which either retard or hasten tumour development have been described; these have been reviewed by Orr (1947). It must be stressed, however, that no agent has thus far been described which will completely inhibit hepatic tumour development provided the carcinogen is administered long enough.

In previous experiments (Wyatt and Howell, 1953) an unsuccessful attempt was made to produce pigmentary cirrhosis in rats maintained on a corn-grit diet supplemented with copper acetate and ferric citrate. As DMAB is an agent known to be capable of inducing cirrhosis it was decided to repeat these experiments including DMAB in the diet. The results of this experiment are now reported since it became apparent that copper acetate had a marked inhibitory effect on hepatic tumour development and cirrhosis.

### MATERIALS AND METHODS

### *General*

The experiments were carried out in two series. In Experiment A the rats were derived from entirely out-bred laboratory stock, in Experiment B, rats from a heterozygous strain were employed (Laboratory Animals Bureau Catalogue of Uniform Strains, No. 626, 1953 ; the Birmingham strain).

In both experiments the animals, whose age varied between 2 and 6 months at the start of the experiment, were kept in galvanised wire mesh cages, never more than 5 rats to a cage. The various diets were moistened with water to prevent the scattering of feed, and placed in galvanised troughs in the cage every morning. The amount of food given was calculated on the basis of 10 g. per rat per day. Water was available *ad lib.* In Experiment B, to check the quantity of food consumed by the rats in each cage, at certain times the residue was dried and weighed.

After the third month of treatment all the animals were examined at approximately 14 day intervals to determine the presence of palpable liver tumours. In Experiment A, animals with tumours were allowed to live their full term, or until the tumour became so large as to be incapacitating. In Experiment B the animals were usually killed as soon as, or shortly after, a tumour had become palpable.

*Composition and preparation of diets*

Two basic forms of diet were used : finely ground maize (obtained from a local dealer) was substituted for corn-grits, since these were unobtainable locally and rat cube powder. The latter was derived from rat cubes (Heygate and Sons, known as the Thompson diet). The Thompson diet is well balanced and has been found to maintain normal growth and nutrition. The biochemical composition of maize varies from season to season. It is very rich in nitrogen-free extract, mainly starch, and has a relatively high fat content. It never contains more than 10 per cent protein, principally zein, which is of poor biological value, and is deficient in two essential amino acids : lysine and tryptophane. Attention has been drawn to the badly balanced character of the amino acids present in zein (Gillman and Gilbert, 1954). The ergosterol, niacin, choline, riboflavin, calcium and phosphorus content is low.

Maize is of poor nutritional value for rats and when it is used as the sole source of food growth is retarded. Even when it is supplemented with lysine and tryptophane growth is still retarded and its protein value is not equivalent to that of casein (Hogan *et al.*, 1955).

The maize and cube powder were weighed and dry crystalline DMAB was added to a concentration of 0·09 per cent. Powdered copper acetate and/or ferric citrate were added in concentrations of 0·5 per cent and 2·0 per cent respectively. The diets were shaken until an intimate admixture was obtained and were stored in enamel bins. They were freshly prepared at approximately 10 day intervals.

*Dietary groups*

*Experiment A.*—Forty rats were divided into 4 groups, with equal numbers of males and females in each group. The appropriate diet for each group (Table I) was given continuously throughout the life of the animals. When the results of this experiment were analysed and it had become apparent that some factor, presumably copper acetate, was affecting the carcinogenic process it became necessary to determine whether or not in fact the copper salt was the inhibiting agent and that the inhibition was not due to the combined effect of the copper and iron salts. Furthermore, the possibility that copper interfered with the intestinal absorption of DMAB, or even combined with it in the intestinal tract, had to be considered. Accordingly, a second experiment was arranged.

TABLE I.—*Experiment A* (*Groups* 1, 2, 3 *and* 4). *Diets*

| Group | Number of rats: Male | Female | Diet |
|---|---|---|---|
| 1 | 5 | 5 | CP + Fe cit + DMAB. |
| 2 | 5 | 5 | M + Fe cit + DMAB. |
| 3 | 5 | 5 | CP + Fe cit + Cu ac + DMAB. |
| 4 | 5 | 5 | M + Fe cit + Cu ac + DMAB. |

CP = Cube powder, M = Maize, Fe cit = 2·0 per cent Ferric citrate, Cu ac = 0·5 per cent Copper acetate, DMAB = 0·09 per cent *p*-Dimethylaminoazobenzene.

*Experiment B.*—Seventy rats were divided into 7 dietary groups with equal numbers of males and females in each group. To confirm that copper acetate was the inhibiting agent 3 dietary groups were arranged (Table II). The other dietary groups in this experiment were designed to reduce the likelihood of copper acetate interfering with DMAB absorption or of combining chemically with it in the gut. For this purpose a system of alternating feeding was devised in which the copper acetate and DMAB components of the diet were given on different days (Tables III and IV). An additional group was included to compare the protective effect of copper acetate with the protection afforded by a normal diet given on alternate days.

TABLE II.—*Experiment B* (*Groups* 5, 6 *and* 7). *Diets*

| Group | Number of rats: Male | Female | Diet |
|---|---|---|---|
| 5 | 5 | 5 | M + DMAB. |
| 6 | 5 | 5 | M + DMAB + Cu ac. |
| 7 | 5 | 5 | M + DMAB + Fe cit. |

M = Maize, DMAB = 0·09 per cent *p*-Dimethylaminoazobenzene, Cu ac = 0·5 per cent Copper acetate, Fe cit = 2·0 per cent Ferric citrate.

TABLE III.—*Experiment B* (*Groups* 8, 9, 10 *and* 11). *Diets : DMAB and Copper Acetate Components Given Separately*

| Group | Number of rats: Male | Female | Diet | Number of days per week |
|---|---|---|---|---|
| 8 | 5 | 5 | M + DMAB + Fe cit | 4 |
| | | | M + Cu ac | 3 |
| 9 | 5 | 5 | M + DMAB + Fe cit | 5 |
| | | | M + Cu ac | 2 |
| 10 | 5 | 5 | M + DMAB + Fe cit | 6 |
| | | | M + Cu ac | 1 |
| 11 | 5 | 5 | M + DMAB + Fe cit | 4 |
| | | | CP | 3 |

M = Maize, CP = Cube powder, Fe cit = Ferric citrate, Cu ac = Copper acetate, DMAB = 0·09 per cent *p*-Dimethylaminoazobenzene.

TABLE IV.—*Experiment B* (*Groups* 8, 9, 10 *and* 11).
*To Show Days of Week on Which DMAB or Copper Acetate Was Given*

| Group | Monday | Tuesday | Wednesday | Thursday | Friday | Saturday | Sunday |
|---|---|---|---|---|---|---|---|
| 8 | Cu ac | DMAB | Cu ac | DMAB | Cu ac | DMAB | DMAB |
| 9 | ,, | ,, | DMAB | ,, | ,, | ,, | ,, |
| 10 | DMAB | ,, | ,, | ,, | ,, | ,, | ,, |
| 11 | CP | ,, | CP | ,, | CP | ,, | ,, |

Basal diet maize, except where CP is indicated.

*Special procedures*

In Experiment A rats were subjected to liver biopsy under anaesthesia. They were biopsied in rotation at monthly intervals, from the second to the tenth months of the experiment. During Experiment B, liver function was assessed by means of the bromsulphalein excretion test.

In Experiment B the animals were weighed regularly, and it was found that all animals gained weight, although at a slower rate than normal. During the first month of the experiment animals in Group 6 showed a loss of weight which was later slowly regained. This initial loss was thought to be due to abstention from food. Animals of this group were all heavier than animals of other groups and hence were better able to undergo voluntary starvation rather than consume an unpalatable diet.

At intervals, over periods of one month the quantity of food consumed by the animals was estimated by weighing the residues daily. The results obtained are only a rough guide to the quantity of food consumed since no allowance was made for spillage or for individual variations in consumption, but it was found that the animals in the various dietary groups consumed roughly the same amounts of food.

It was essential to determine whether or not DMAB underwent any chemical alteration when it was mixed and stored with maize and copper acetate. For this purpose column chromatography was undertaken on diets which had been prepared and stored for about 2 months prior to the test. Columns were prepared from a slurry of alumina (B.D.H. activated), ligroin extracts of two diets, one consisting of maize and 0·09 per cent DMAB, the other of maize, 0·09 per cent DMAB and 0·5 per cent copper, were dropped on the columns and washed through with suitable solvents. These two columns were compared with similarly prepared columns of DMAB alone, and of a mixture of DMAB and 4-aminoazobenzene. A clear separation of the two components of the last column was obtained, DMAB flowing faster than 4-aminoazobenzene. The columns containing extracts of the diets gave only one component corresponding to DMAB. Further experiments were undertaken to determine if there was any reduction in the quantity of DMAB in the prepared stored diets. The dye was extracted from the diet and estimated colorimetrically on the day the diet was prepared and thereafter at weekly intervals for 6 weeks. No decrease in the amount of dye was detected during this period.

*Post-mortem examination and histological methods*

Blocks of tissue from the liver and spleen were preserved for microscopic study, together with any other tissue which showed pathological changes. At least two blocks were taken from every liver ; frequently, and especially when more than one tumour was present, several blocks were taken, although no attempt was made to section every tumour.

The tissue was fixed in 4 per cent formaldehyde-saline or occasionally in Bouin's fluid. Sections were stained with Ehrlich's haematoxylin and eosin, Weigert's haematoxylin and Van Gieson, and by the periodic-acid-Schiff method using a diastase-treated control. Perls' reaction for ferric iron was carried out on all sections. Other stains used, included Gomori's reticulin method and Best's carmine stain for glycogen. Frozen sections were cut and stained for fat. Special histochemical stains for copper were also employed.

### RESULTS

The first animal to develop tumours in both Experiment A and B died during the sixth month. Hence this period has been taken as the minimum induction period and animals that died before this time have been excluded since they were not " at risk ". The results in the two experiments are detailed in Tables V and VI.

TABLE V.—*Experiment A. Details of Tumour Incidence*

| | Group | | | |
|---|---|---|---|---|
| | 1 | 2 | 3 | 4 |
| Animals " at risk " | 10 | 9 | 8 | 8 |
| Months to first tumour | 9 | 6 | 11 | No tumour |
| Other animals alive at time of first tumour | 9 | 8 | 5 | — |
| Number developing tumours | 8 | 8 | 4 | 0 |
| Average induction time and range (months) | 13·6 (9–16) | 11·25 (6–16) | 14·0 (11–16) | — — |
| Average time to death and range (Months) | 13·0 (9–16) | 11·0 (6–16) | 12·5 (8–16) | 13·5 (10–16) |

Animal " at risk " = animals surviving more than 6 months.

TABLE VI.—*Experiment B. Details of Tumour Incidence*

| | Group | | | | | | |
|---|---|---|---|---|---|---|---|
| | 5 | 6 | 7 | 8 | 9 | 10 | 11 |
| Animals " at risk " | 8 | 8 | 10 | 10 | 8 | 10 | 9 |
| Months to first tumour | 6 | 18 | 6 | 15 | 9 | 9 | 15 |
| Other animals alive at time of first tumour | 7 | 0 | 9 | 7 | 5 | 9 | 5 |
| Number developing tumours | 8 | 1 | 10 | 3 | 6 | 8 | 5 |
| Average induction time and range (Months) | 8·5 (6–10) | — — | 8·5 (6–12) | 15 — | 11·3 (9–15) | 12 (9–15) | 16·2 (15–18) |
| Average time to death and range (Months) | 8·5 (6–10) | 13·3 (10–18) | 8·5 (6–12) | 15·1 (8–19) | 10·5 (8–15) | 11·4 (9–15) | 14·1 (9–18) |

Animals " at risk " = animals surviving more than 6 months.

*Experiment A* (Table V)

*Group* 1.—Ten animals survived 6 months, of which 8 developed tumours. The first tumour was found at 9 months, at which time 9 other animals were alive. Other tumours appeared between 9 and 16 months (average 13·6 months). The 2 rats without tumours died after 9 and 12 months' treatment.

*Group* 2.—Nine animals survived 6 months, of which 8 developed tumours. The first tumour was found at 6 months, at which time 8 rats were alive. Other tumours appeared between 6 and 16 months (average 11·25 months). The animal without a tumour died at 9 months as a result of liver biopsy.

*Group* 3.—Eight animals survived 6 months, but only 4 developed tumours. The first tumour was found at 11 months, at which time 5 rats were surviving. Other tumours appeared between 11 and 16 months (average 14 months). The 2 animals without tumours died during the eleventh and fourteenth months of treatment.

*Group* 4.—No animal developed a tumour in this group although 8 survived treatment for 6 months. They died between 10 and 16 months (average 13·5 months).

*Comment.*—In Groups 1 and 2 combined, only 3 animals died without tumours. However, there was a difference between the two groups of 2·3 months in the average tumour induction time. This difference may indicate a protective effect of rat cube due to its greater nutritional value as compared with maize.

When copper acetate was given with the carcinogen in a maize diet (Group 4) no tumours were found despite the fact that the group survived for an average period of 13·5 months, i.e. 2·25 months longer than the corresponding group without copper (Group 2). With a cube and copper diet (Group 3), 4 of a possible 8 animals developed tumours, the average time of tumour development being 14 months. Although this is only 0·4 months longer than in the corresponding group without copper (Group 1), the first tumour in Group 3 did not appear until the eleventh month, whereas the first tumour in Group 1 appeared during the ninth month. In Group 1, furthermore, 8 animals out of a possible 10 developed tumours.

It is apparent from these results that diets containing ferric citrate and copper acetate, especially when incorporated into a maize diet, afford a considerable degree of protection against hepatic tumour development. When given in conjunction with a rat cube diet the protection is not so marked. Chemical analyses suggest that copper storage in the liver is greater in maize-fed animals than in cube-fed animals. This might be an important factor in determining the difference in tumour yield between the two basal diets.

*Experiment B* (Table VI)

*Groups* 5 *and* 7 may be considered together. In both groups the first tumours developed during the sixth month, and all the animals "at risk" developed tumours. The average time to death in both groups was 8·5 months.

*Group* 6.—Eight rats survived 6 months. One tumour was found during the eighteenth month in the sole survivor of the group. The other 7 animals had died in 10–15 months (average 13·3 months).

*Group* 8.—All 10 rats survived treatment for 6 months. Three tumours developed in the fifteenth month. The remaining 5 animals died between 16 and 19 months without tumours.

*Group* 9.—Eight animals survived 6 months' treatment, 6 of which subsequently developed tumours, the first being found after 9 months. The average time to death in tumour bearing animals was 11·3 months. The 2 animals without tumours died during the eighth month.

*Group* 10.—Ten animals survived 6 months' treatment, of these, 8 subsequently developed tumours, the first being found during the ninth month. The other tumours were found between 11 and 15 months, and the average time to death due to tumour development was 12 months.

*Group* 11.—Nine animals survived 6 months' treatment, of which 5 developed tumours. The first tumours, 3 in number, were found after 15 months. The other 2 tumours appeared at 18 months. The average time to death due to tumour development was 16·2 months. Animals died between the ninth and sixteenth months, but none had tumours.

*Comment*.—There is a difference in the time of death due to tumour development in Experiments A and B when groups comparable, except for the addition of ferric citrate, are considered. This difference arises from differences in experimental technique. In Experiment A the animals were allowed to live until moribund or until they died as a result of tumour growth. Liver biopsies were done in strict rotation and no special effort was made to biopsy those suspected of having tumours. During Experiment B, when the animals were submitted to bromsulphalein excretion tests, careful abdominal palpation was carried out under anaesthesia. An effort was made to test all the animals suspected of having tumours as soon as they developed, and with certain exceptions they were then killed. In this way tumours were found at an earlier stage of development than in Experiment A. In addition, since there is some evidence that strains differ in their susceptibility to the carcinogen (Sugiura and Rhoads, 1941), part of the difference in average induction times may be due to the different strains of rat used in the two experiments.

In Experiment B it has been confirmed that copper acetate is a powerful tumour inhibiting agent and that ferric citrate is without effect on the carcinogenic process. In Group 6 receiving copper acetate, none of the 8 animals at risk died before the tenth month, by which time all the animals of Group 5 had developed and died with tumours. The solitary tumour in Group 6 did not appear until the eighteenth month. The average time to death for this group, excluding the animal with a tumour, was 12·7 months, i.e. 4·2 months longer than in Group 5.

The results of the alternating feeding experiments are more difficult to interpret since it is not certain how many of the effects observed were due to the reduced DMAB consumption or to the supplementary copper. Groups 8 and 11 may be compared. In both, tumours developed in several animals during the fifteenth month of treatment; excluding these animals there were 5 survivors in Group 8 and 3 survivors in Group 11. None of the survivors in Group 8 developed tumours even though 3 of them lived to the nineteenth month of treatment; but 2 of the 3 survivors in Group 11 developed tumours during the eighteenth month. Considering the nature of the basal diet of these groups, the expectation of tumour development is greater in Group 8, which received maize every day, than in Group 11, which received cube or maize on alternate days. Despite this the tumour incidence was less in the copper-treated group. It would thus appear that the inhibitory effect of copper feeding is just apparent in Group 8.

When Group 8 is compared with Groups 9 and 10 it can be seen that tumour inhibition is much less in the two latter groups. In Groups 9 and 10 the first tumours appeared during the ninth month and all the animals dying subsequently had tumours. The prolongation of the time to the first appearance of tumours in these two groups, together with the prolongation of the average induction times when compared with Group 7, appear greater than can be accounted for by the omission of DMAB from the diet for one or two days of each week and may be due in part to the addition of copper acetate to the diet.

### PATHOLOGY

*Biopsy material* (Experiment A)

Histological assessment of the tissue obtained from 33 animals between the second and tenth months of dietary treatment allows comparisons to be made between animals of the 4 dietary groups. However, since only one specimen of liver is available from each group per month, it cannot be assumed that the changes in that one specimen are representative of the whole group at that particular time. Nevertheless, the material does reveal differences among the dietary groups, both in severity and rate of development of lesions.

The early microscopical changes which follow the administration of DMAB have been described by Orr (1940), and Opie (1944) and a detailed description is unnecessary. In general the effect of DMAB is to cause the gradual piecemeal destruction of liver cells, at first in a zone adjacent to portal tracts and later throughout the lobule. This is followed by regenerative hyperplasia without normal lobular architecture. This may consist of intralobular disorganisation in which foci of regenerative cells within a lobule show a derangement of the normal radial pattern of liver cell cords, frequently with signs of pressure distortion of surrounding liver cell cords and displacement of the central vein of the lobule (Fig. 1). Regenerative hyperplasia may progress to nodular hyperplasia in which varying sized nodules of regenerating liver cells are formed without normal lobular architecture (Fig. 2). These nodules are often formed from the remains of several liver lobules. Concurrently with these changes, portal tracts show infiltration with chronic inflammatory cells and macrophages (Fig. 3) At first confined to portal tracts, these cells extend into adjacent parts of the liver lobule and towards other portal tracts. They are accompanied by reticulin fibres (Fig. 4) which are gradually superimposed by collagen and eventually a multilobular cirrhosis is produced (Fig. 2). Bile duct proliferation and dilatation also occurs, proliferation is first seen in the form of double rows of fusiform cells with poorly defined cell borders and large oval nuclei, poor in chromatin. These cells accompany the chronic inflammatory cells as they extend from the portal tracts (Fig. 5 and 6).

The development of cirrhosis and regenerative hyperplasia form a convenient means of assessing the liver damage. The development of cirrhosis can be arbitrarily divided into 3 grades : periportal fibrosis or incipient cirrhosis, and early and advanced cirrhosis. Periportal fibrosis (incipient cirrhosis) is a very early stage, in which the newly formed fibrous tissue is either confined to the portal tracts or at the most, thin strands of fibrous tissue extend only a little way into the adjacent parenchyma (Fig. 7). Macroscopically the liver is always smooth.

Early cirrhosis is a later stage, in which thin strands of fibrous tissue extend a considerable way around liver lobules and pseudo-lobules (Fig. 8). Slight granularity may or may not be seen on macroscopic examination.

In advanced cirrhosis the fibrous tissue is denser and the strands thicker than that described above. The strands frequently completely encircle the lobules and regeneration nodules, and the appearances resemble human multilobular, Laennec-type cirrhosis (Fig. 2). At this stage the liver is always macroscopically granular.

In Table VII is given the detailed incidence of the various grades of cirrhosis assessed on the maximum seen in any section, together with the presence or absence of regenerative hyperplasia of liver tissue in the 4 dietary groups. In the material from 8 rats of Group 4 no evidence of cirrhosis or of hyperplasia was seen. Material from a similar number of rats is available from Group 1, 5 of which showed varying grades of cirrhosis ; in 4 regenerative hyperplasia was present. In only 3 animals was cirrhosis absent. In Group 3, material was available from 9 rats ; 3 showed regenerative hyperplasia and only one showed evidence of cirrhosis. From Group 2, 8 rats were subjected to biopsy ; 6 of them revealed regenerative hyperplasia, and 7 showed some degree of cirrhosis. In only one animal was cirrhosis absent.

TABLE VII.—*Experiment A. Number of Biopsies in Each Group Related to the Number of Animals Showing Regenerative Hyperplasia and Cirrhosis of the Liver*

| Group | Number of biopsies | Regenerative hyperplasia | Degree of cirrhosis: Absent | Incipient | Early | Advanced |
|---|---|---|---|---|---|---|
| 1 | 8 | 4 | 3 | 2 | 3 | — |
| 2 | 8 | 6 | 1 | 3 | 3 | 1 |
| 3 | 9 | 3 | 8 | — | 1 | — |
| 4 | 8 | — | 8 | — | — | — |

It is apparent that liver damage was considerably less in Group 4 than in the other 3 groups. In this group the histological changes appeared to be arrested, at least during the period under study, following the development of increased periportal cellularity associated with fusiform cell proliferation. Liver cell damage was nearly always confined to cells adjacent to portal tracts. Nevertheless, it must be stressed that the histological changes in this group were all of the type which follow DMAB administration.

From the biopsy material an estimate can also be made of the rate of development of hepatic damage. Thus, in Group 1 the specimen obtained at 3 months showed early cirrhosis. In the specimen at 4 months the liver showed incipient cirrhosis with regeneration and a marked tendency for fusiform cells to encircle hepatic lobules. Similar processes were observed in all the other animals studied ; a tumour was found during the ninth month.

In Group 2, at 2 months a very severe degree of liver damage was found with early cirrhosis and nodular hyperplasia. It seems most likely that the severity of the lesions was exceptional in the particular rat examined. In the following months the specimens all showed some degree of cirrhosis with regenerative hyperplasia. At 6 months a tumour was found.

It was not until the fourth month that pronounced changes were observed in Group 3 rats, even then the animal appeared to be an exception in that the liver showed early cirrhosis and nodular hyperplasia. No cirrhosis was found in this group subsequently, and further, although at 6 and 7 months intralobular disorganisation was present, specimens at 8 and 9 months showed only minimal changes.

In Group 4 it was not until the eighth month that marked changes were seen, consisting of fusiform cells and chronic inflammatory cells tending to encircle lobules.

From these observations it is apparent that hepatic injury developed and progressed rapidly in Group 2 rats, followed closely by rats from Group 1. Group 4 animals showed a very considerable retardation in the severity of the lesions and a considerable prolongation in the time required to produce them.

*Histological changes in animals dying without tumours*

An assessment of the changes seen in these animals is included since they supplement the observations made on the biopsy material and help to evaluate still further the protective effect of copper acetate.

In Experiments A and B combined 37 animals failed to develop tumours after surviving a minimum of 6 months' treatment with DMAB. With the exception of Groups 5 and 7, every group contained one or more animals without tumours.

The tumourless animals can be divided into 3 groups : those that did not receive copper acetate, those that received copper acetate continuously, and those that received copper acetate on certain days of the week only. The assessment of liver damage in these groups can be based on the criteria used for the assessment of the biopsy material. The detailed results are set out in Table VIII.

TABLE VIII.—*Cirrhosis in Animals Without Tumours in Experiments A and B*

| Group | Animals "at risk" | Animals without tumours | Regenerative hyperplasia | Degree of cirrhosis: Absent | Incipient | Early | Advanced |
|---|---|---|---|---|---|---|---|
| 1 | 10 | 2 | 2 | — | — | 2 | — |
| 2 | 9 | 1 | 1 | — | 1 | — | — |
| 3 | 8 | 4 | — | 2 | 2 | — | — |
| 4 | 8 | 8 | 4 | 3 | 4 | — | 1 |
| 6 | 8 | 7 | 5 | 2 | 3 | 1 | 1 |
| 8 | 10 | 7 | 5 | 2 | 2 | 3 | — |
| 9 | 8 | 2 | 2 | — | 2 | — | — |
| 10 | 10 | 2 | 2 | 1 | 1 | — | — |
| 11 | 9 | 4 | 1 | 4 | — | — | — |

Animals "at risk" = animals surviving more than 6 months.

Only 3 animals given DMAB without copper acetate (Groups 1 and 2) did not develop tumours. Regenerative hyperplasia associated with incipient or early cirrhosis was present in all 3.

Groups 3, 4 and 6 received DMAB and copper acetate continuously, and a total of 19 animals did not develop tumours. Regenerative hyperplasia was present in 9 of these animals ; incipient cirrhosis was observed in 9, early cirrhosis in one, and advanced cirrhosis in 2. Cirrhosis was completely absent in 7. After the thirteenth month all the animals dying in these groups had developed incipient cirrhosis and intralobular disorganisation.

There were 15 animals without tumours in the remaining groups receiving DMAB and copper acetate separately. In 10 of these, regenerative hyperplasia was observed ; incipient cirrhosis was present in 5 and early cirrhosis in 3. No animal showed advanced cirrhosis, and in 7, cirrhosis was completely absent.

These observations show quite clearly that the microscopical changes in the DMAB, copper-treated rats are of the same nature as those which result from DMAB alone, but there is a delay in the rate of development and progression of the lesions especially marked during the first 12 months of treatment. Many of the animals receiving DMAB and copper eventually developed varying grades of cirrhosis, the incipient type predominating. In some animals this was accompanied by regenerative hyperplasia, frequently associated with bile duct proliferation and dilatation ; but by the time these changes had developed, all the animals treated with DMAB alone had developed and died with tumours. Nevertheless, the impression is gained from the histological material that had the copper-treated animals survived, and had treatment been continued long enough, some tumours would eventually have been produced. In fact one tumour did develop during the eighteenth month of treatment in a rat from Group 6.

*Tumours and cirrhosis*

Two of the tumours produced by DMAB are derived from bile duct epithelium, they are the bile duct cystadenoma and the cholangiocarcinoma ; a third tumour is derived from liver cells, the hepatoma. The gross and microscopic features of these tumours have been described in detail by numerous workers and since the tumours produced in the present experiments did not differ in any way from those previously described a detailed account is unnecessary. The lesion known as cholangiofibrosis also arises from bile duct epithelium and must be considered a premalignant lesion because in common with Opie (1944) and Firminger (1955) I have observed transitions between this lesion and cholangio carcinoma.

In Experiments A and B combined, a total of 61 animals developed tumours. In 41 of these animals the tumours were of 2 or more histological types, and were frequently intimately admixed. In the remaining 20 only one tumour was present in each liver, the tumour varying in histological type. This incidence of multiple tumours in the liver has been noted previously by several workers. The tumours which occurred in the animals receiving different amounts of copper in the diet did not exhibit any unusual features.

Macroscopic cirrhosis usually accompanies the appearance of DMAB induced hepatic tumours, but it is not an essential precursor (Miller *et al.*, 1941 ; Opie, 1944 ; Hoch-Ligeti, 1946). However, in the presence of cirrhosis the tumour yield is increased and the induction time decreased. In the present experiments a portion of non-tumorous liver tissue was taken for histological examination from all the animals with hepatic tumours. The degree of cirrhosis was assessed in the same manner as in the biopsy material. In the 61 animals with tumours, cirrhosis was absent in only 4 instances. Incipient cirrhosis was present in 12, early cirrhosis in 22 and advanced cirrhosis in 23. These figures show the close association between the development of hepatic tumours and the presence of cirrhosis. They can be contrasted with the incidence and degree of cirrhosis in the 37 non-tumour bearing animals in which cirrhosis was absent in 14, incipient in 15, early in 6, and advanced in 2.

*Splenic changes*

Splenic enlargement, developing early in the course of DMAB carcinogenesis, was described by Orr (1940). This enlargement was present before the liver had developed microscopical changes and became maximal during the third and fourth months of treatment, thereafter tending to shrink. Enlargement of the spleen was also observed by Edwards and White (1941) and Hoch-Ligeti (1946).

During Experiment B all spleens were weighed at post-mortem examination. In Groups 5 and 7 receiving DMAB with or without ferric citrate but no copper acetate, the mean splenic weight in the 2 groups was approximately equal (2·3 g. and 2·2 g. respectively), and approximately 1·0 g. heavier than the mean weight of the spleen in Group 6 (1·1 g.), which received DMAB and copper acetate. The difference in the splenic weights of the directly comparable groups, 5 and 6, was significant ($0{\cdot}02 > P > 0{\cdot}01$).

In the other part of Experiment B where the diets were alternated, Groups 9 and 10 had the largest spleens ; these groups received the smallest quantities of copper and the largest quantity of DMAB. In Groups 8 and 11 the mean weights of the spleen were approximately equal, but the mean weight of both was greater than in Group 6.

As the spleen enlarges during DMAB treatment its normally sharp edges become rounded, the colour becomes darker; later the surface is dulled and shows slight granularity with pitting. The consistency is much firmer than normal.

Microscopically, the spleen is congested at first, later varying degrees of fibrosis develop. The fibrous trabeculae may become much more prominent and thicker than normal (Fig. 9). In some spleens fibrous tissue is formed in the Malpighian bodies which may become almost completely replaced by collagen (Fig. 10). In this type of fibrosis many Malpighian bodies are usually involved. Another variant is where fibrous tissue is present in rather localised areas in the pulp thickening the walls of the sinusoids (Fig. 11).

These gross and microscopic changes, which are essentially the same as those described by Orr (1940), are also observed in copper acetate treated rats, but the changes in these animals were always much less advanced than in those animals given DMAB without copper acetate.

No correlation was observed between the weight of the spleen and any of the following changes in the liver: liver weight, tumour type, the presence or absence of cirrhosis or regenerative hyperplasia. In certain animals splenic enlargement and congestion were observed without marked pathological change in the liver, and splenic fibrosis was observed without cirrhosis. These observations suggest that the splenic changes are not due to vascular obstruction in the liver, with consequent portal hypertension.

### DISCUSSION

There are other reports in the literature dealing with the effect of copper on azo-dye carcinogenesis which show that the protection is probably independent of the type of salt used, since both copper acetate and copper sulphate have been found to be effective. Sharpless (1946) in a study of trace-elements and azo-dye carcinogenesis used low-riboflavin diets containing DMAB supplemented by 0·15, 0·3 and 0·5 per cent copper sulphate. No effect was observed on tumour incidence but the induction time was increased by 25–50 per cent as compared to that observed with the control diets. No other details are given in this short report, except that the non-malignant liver damage was always less than in the controls.

During the early stages of the present experiments Pedrero and Kozelka (1951) added copper (the salt used was not stated) to a diet containing the carcinogen 3′-methyl-4-dimethylaminoazobenzene which is structurally related to DMAB. The concentration of copper was either 0·25 or 0·5 per cent. The experiments were terminated after treatment for 6 months. The control group receiving the carcinogen without copper contained 30 animals of which 27 developed tumours. The diet containing 0·25 per cent copper was given to 20 rats and only 12 of these developed tumours. The first tumours in both these groups were found during the third month. The diet containing 0·5 per cent copper proved toxic and only 5 animals survived 3 months' treatment, but the liver of all these proved normal on macroscopic and microscopic examination. Why this level of copper proved toxic is not clear, except that the carcinogen used is more powerful than DMAB and the basal diet was even less nutritionally adequate than maize. They make no mention of a toxic effect due to this level of copper in control animals given a similar diet without the carcinogen.

Clayton, King and Spain (1953) used copper-free diets and diets containing either 3·94 or 300 mg./kg. of copper sulphate. Both DMAB and 3′-methyl-4-dimethylaminoazobenzene were used and with both there was definite protection against tumour development when the higher concentration of copper was given. The incidence and severity of cirrhosis was also less in the copper-treated animals. Actual numbers of animals and times of tumour development are not given in this short paper.

In a recent paper King, Spain and Clayton (1957), whilst observing the inhibitory effect of copper acetate on tumour development due to 3′-methyl-4-dimethylaminoazobenzene, also noted that the addition of copper sulphate to the synthetic diet they used caused rapid destruction of the carcinogen. They also observed that their diet, which contained 79 per cent glucose and a considerable quantity of fat, frequently became rancid when copper sulphate was mixed with it. They concluded that much of the inhibitory effect of the copper salt on tumour development was due to destruction of the carcinogen. Nevertheless, they were still able to obtain inhibition of tumours when the diet was freshly prepared every day. Rancidity was never observed in any of the diets used in the present experiments. Because the diets were freshly prepared at approximately 10 day intervals and since no evidence of destruction of dye in the diets has been found over periods of 6 weeks, it is considered that destruction of the dye can be excluded as a factor in the retardation of tumour development in the present experiments.

The mechanism whereby copper exerts its protective effect must at present be a matter for conjecture. There are a number of possibilities. Groups 8, 9, 10 and 11 of Experiment B were designed to reduce the likelihood of a chemical alteration of the carcinogen under the influence of copper acetate in the gastro-intestinal tract. The results obtained from these groups are inconclusive since the tumour incidence and induction times were very different from those in the control group receiving DMAB every day. Analysis of the histological material from Groups 4 and 6 receiving copper and DMAB continuously show that the changes in the liver were similar to those which follow DMAB alone, but less severe. These observations suggest that DMAB in an active form was reaching the liver and that any chemical alteration or interference with absorption in the gut was incomplete.

Kensler, Sugiura and Rhoads (1940) showed that DMAB treatment causes reduction in the riboflavin content of the liver ; Griffin and Baumann (1946) showed that the reduction of riboflavin in the liver was directly related to the carcinogenicity of the compound tested. Later Griffin and Baumann (1948) were able to show that hydrogenated coconut oil, a protective agent, maintained the riboflavin content of the liver when DMAB was included in the diet. Clayton, King and Spain (1953) reported that copper feeding, regardless of the simultaneous administration of the carcinogen, actually increased the riboflavin content of the liver. King, Spain and Clayton (1957) using diets containing 3′-methyl-4-dimethylaminoazobenzene and copper sulphate freshly prepared each day have reported that the riboflavin content of the liver did not fall so much as when the diet contained no copper. These observations suggest that the mechanism of protection is similar to that of high protein diets and hydrogenated coconut oil, and is mediated through maintenance of the riboflavin content of the liver.

The importance of binding of the active carcinogenic derivative of DMAB to liver proteins has been stressed by Miller and Miller (1955). The major portion of the bound dye, which is found only in the liver, is associated with some of the soluble proteins. Copper has the ability to complex very readily with a wide variety of proteins, and copper salts have been described as being " protein-avid ". King *et al.* (1957) estimated the total and bound dye in the liver of their copper-carcinogen treated animals and found that they did not increase as rapidly as in those animals not receiving copper. It is not inconceivable that the metal competes with the active portion of DMAB for binding to protein in the liver, and so prevents or delays the changes in the protein content of the hepatic cells which Miller and Miller (1955) envisaged as leading to tumour formation.

Finally there are certain physiological properties of copper through which the protective effect might be mediated. Copper is concerned with glucose metabolism (Keil and Nelson, 1934). It forms an essential part of butyryl-coenzyme A (Gubler, 1956) which catalyses the first step in the oxidation of short-chain fatty acids with 3–8 carbon atoms. Copper is aiso concerned in purine metabolism by the copper-containing enzyme, uricase. There is evidence that copper is required for the activation of the cytochrome reactions and energy transfer of cells. Copper is also moderately bactericidal and it is possible that ingestion of high concentrations produces an alteration in the bacterial flora of the bowel, so that vitamin synthesis in the bowel is altered and the balance upset. Vitamins of the B group are known to be of importance in hepatic tumour development, some accelerating tumour development, others retarding it.

Thus it can be seen that copper could alter azo-dye carcinogenesis by a variety of means, and since it is an essential part of many enzyme and bio-synthetic reactions it is conceivable that enzymes and reactions which involve copper may be altered during carcinogenesis and that a high copper diet modifies this. Alternatively a high copper diet and the consequent tissue storage may itself inactivate some enzyme system or systems which are involved in the metabolism of the dye and so prevent tumour development.

### SUMMARY

Experiments are described which show that copper acetate has a powerful retarding effect on hepatic tumour development in rats treated with DMAB. Thus, of 16 rats which survived treatment with copper acetate and DMAB for longer than 6 months, only one animal developed a tumour which was found after 18 months' treatment. This can be contrasted with a control group receiving DMAB alone, in which 8 rats all developed tumours in an average time of 8·5 months.

An assessment of liver damage based on the development of cirrhosis and regenerative hyperplasia is described. From the analysis of liver tissue obtained by biopsy during the experiment, and from animals dying without tumours, it has been found that liver damage and cirrhosis is always much less in copper acetate-DMAB treated rats than in rats receiving DMAB alone. The spleen of the copper-treated rats also shows less damage and does not enlarge so much as in the group treated with DMAB alone.

Possible mechanisms through which the protective effect might be mediated are briefly discussed. It has been established that there is no alteration or destruction of the carcinogen when mixed and stored with copper acetate.

I am indebted to Professor J. W. Orr and Dr. D. L. Woodhouse for valuable help and advice throughout this investigation. I am also indebted to the Birmingham Branch of the British Empire Cancer Campaign and to the Medical Research Fund of the United Birmingham Hospitals for support of this work.

### REFERENCES

Clayton, C. C., King, H. J. and Spain, J. D.—(1953) *Fed. Proc.*, **12**, 190.
Edwards, J. E. and White, J.—(1941) *J. nat. Cancer Inst.*, **2**, 157.
Firminger, H. I.—(1955) *Ibid.*, **15**, 1427.
Gillman, G. and Gilbert, C.—(1954) *Ann. N.Y. Acad. Sci.*, **57**, 737.
Griffin, A. C. and Baumann, C. A.—(1946) *Arch. Biochem.*, **11**, 467.—(1948) *Cancer Res.*, **8**, 279.
Gubler, C. J.—(1956) *J. Amer. med. Ass.*, **161**, 530.
Hoch-Ligeti, C.—(1946) *Cancer Res.*, **6**, 563.
Hogan, A. G., Gillespie, G. T., Koçtürk, O., O'Dell, B. L. and Flynn, L. M.—(1955) *J. Nutr.*, **57**, 225.
Keil, H. L. and Nelson, V. E.—(1934) *J. biol. Chem.*, **106**, 343.
Kensler, C. J., Sugiura, K. and Rhoads, C. P.—(1940) *Science*, **91**, 623.
King, H. J., Spain, J. D. and Clayton, C. C.—(1957) *J. Nutr.*, **63**, 301.
Kinosita, R.—(1940) *Yale J. Biol. Med.*, **12**, 287.
Miller, E. C. and Miller, J. A.—(1955) *J. nat. Cancer Inst.*, **15**, 1571.
Miller, J. A., Miner, D. L., Rusch, H. P. and Baumann, C. A.—(1941) *Cancer Res.*, **1**, 699.
Opie, E. L.—(1944) *J. exp. Med.*, **80**, 231.
Orr, J. W.—(1940) *J. Path. Bact.*, **50**, 393.—(1947) *Brit. med. Bull.*, **4**, 385.
Pedrero, E. and Kozelka, F. L.—(1951) *Arch. Path.* (*Lab. Med.*), **52**, 455.
Sharpless, G. R.—(1946) *Fed. Proc.*, **5**, 239.
Sugiura, K. and Rhoads, C. P.—(1941) *Cancer Res.*, **1**, 3.
Wyatt, J. P. and Howell, J.—(1953) *Arch. Path.* (*Lab. Med.*), **55**, 466.

---

### EXPLANATION OF PLATES

Fig. 1.—Intralobular disorganisation causing compression and distortion of adjacent sinusoids. H. and V.G. × 125.
Fig. 2.—Nodular hyperplasia and advanced cirrhosis. H. and V.G. × 135.
Fig. 3.—Chronic inflammatory cells and macrophages within a portal tract. Degenerative changes in periportal liver cells. H. and E. × 250.
Fig. 4.—Increased numbers of reticulin fibres radiating from portal tracts. Reticulin. × 140.
Fig. 5.—Fusiform cells extending from portal tract into adjacent parenchyma. H. and E. × 400.
Fig. 6.—Fusiform cells adjacent to a portal tract arranged in double rows. H. and E. × 460.
Fig. 7.—Periportal fibrosis (incipient cirrhosis). Delicate strands of fibrous tissue extending slightly into parenchyma. H. and V.G. × 250.
Fig. 8.—Early cirrhosis. Strands of fibrous tissue extending from portal tracts towards other portal tracts. H. and V.G. × 125.
Fig. 9.—Spleen. Increased prominence of fibrous trabeculae with marked congestion of the pulp. H. and V.G. × 125.
Fig. 10.—Spleen. Fibrosis of Malpighian body. H. and V.G. × 150.
Fig. 11.—Spleen. Fibrosis of pulp. H. and V.G. × 300.